# A practical approach for the validation of sterility, endotoxin and potency testing of bone marrow mononucleated cells used in cardiac regeneration in compliance with good manufacturing practice

**DOI:** 10.1186/1479-5876-7-78

**Published:** 2009-09-08

**Authors:** Sabrina Soncin, Viviana Lo Cicero, Giuseppe Astori, Gianni Soldati, Mauro Gola, Daniel Sürder, Tiziano Moccetti

**Affiliations:** 1 The Cell Therapy Unit, Cardiocentro Ticino, Via Tesserete 48, CH-6900 Lugano, Switzerland

## Abstract

**Background:**

Main scope of the EU and FDA regulations is to establish a classification criterion for advanced therapy medicinal products (ATMP). Regulations require that ATMPs must be prepared under good manufacturing practice (GMP). We have validated a commercial system for the determination of bacterial endotoxins in compliance with EU Pharmacopoeia 2.6.14, the sterility testing in compliance with EU Pharmacopoeia 2.6.1 and a potency assay in an ATMP constituted of mononucleated cells used in cardiac regeneration.

**Methods:**

For the potency assay, cells were placed in the upper part of a modified Boyden chamber containing Endocult Basal Medium with supplements and transmigrated cells were scored. The invasion index was expressed as the ratio between the numbers of invading cells relative to cell migration through a control insert membrane.

For endotoxins, we used a commercially available system based on the kinetic chromogenic LAL-test. Validation of sterility was performed by direct inoculation of TSB and FTM media with the cell product following Eu Ph 2.6.1 guideline.

**Results and discussion:**

The calculated MVD and endotoxin limit were 780× and 39 EU/ml respectively. The 1:10 and 1:100 dilutions were selected for the validation. For sterility, all the FTM cultures were positive after 3 days. For TSB cultures, *Mycetes *and *B. subtilis *were positive after 5 and 3 days respectively. The detection limit was 1-10 colonies.

A total of four invasion assay were performed: the calculated invasion index was 28.89 ± 16.82% (mean ± SD).

**Conclusion:**

We have validated a strategy for endotoxin, sterility and potency testing in an ATMP used in cardiac regeneration. Unlike pharmaceutical products, many stem-cell-based products may originate in hospitals where personnel are unfamiliar with the applicable regulations. As new ATMPs are developed, the regulatory framework is likely to evolve. Meanwhile, existing regulations provide an appropriate structure for ensuring the safety and efficacy of the next generation of ATMPs. Personnel must be adequately trained on relevant methods and their application to stem-cell-based products.

## Introduction

The European Union (EU) regulation on advanced therapy medicinal products [1] (ATMP) is entered into force in all European Member States on December 30, 2008, and Food and Drug Administration (FDA) recently promulgated regulations on human cells, tissues, and cellular and tissue-based products [2] issuing an appropriate regulatory structure for the wide range of stem-cell-based products that may be developed to regenerate damaged tissues. Main scope of the regulations is to establish clear classification criteria for many new cell-based medicinal products. In particular the European Regulation makes reference to and is in coherence with the 2004/23/EC directive on donation, procurement and testing of human cells and tissues and with directive 2002/98/EC on human blood and blood components. This means that any use of human cells has to be in compliance with the quality requirements therein described. The European Regulation is also clear on requiring that all ATMP have to be prepared according to the good manufacturing practice (GMP) for medicinal products. Stem-cell-based therapies have existed since the first successful bone marrow transplantations in 1968 [3]. Among the ATMPs, bone marrow-derived mononuclear cells (BM-MNC), widely used in cellular therapy protocols, include several populations of stem cells able to restore vascularization or to transdifferentiate into functional cardiac cells: hematopoietic stem cells (HSC) which give rise to all mature lineages of blood [4], mesenchymal stem cells (MSC) and endothelial progenitor cells (EPC) which can be mobilized in the peripheral blood and give rise to mature endothelial cells in blood vessels [5]. The hematopoietic lineage is characterized by the presence of the CD34 cell-surface antigen (found in about 1% of human bone marrow mononucleated cells); it has therefore been considered a useful cell selection target for bone marrow progenitor cells. MSC represent less than 0.1% of the bone marrow cell population [6] and are able to generate non hematopoietic tissues including adipocytes, chondrocytes, osteocytes, myocytes [7,8] and cardiomyocites [9]. Angiogenesis and vascuologenesis are responsible for the development of the vascular system and are one of the main mechanisms leading to improved cardiac function after the injection of BM-MNC [10]. Among the CD34+ cells, the CD133 surface antigen defines a subset of hematopoietic stem cells enriched for Endotelial Progenitor Cells (EPCs) [11]. The angiogenic potential of bone marrow cells has been tested into hind limb ischemia animal models [12] and several clinical studies are ongoing to evaluate the efficiency of the intra-arterial administration of BMC into an ischemic limb [13,14].

During the production of the BM-MNC as medicinal products, variable amounts of impurities product and process-related, are introduced into the final product: cells enter in contact with buffers, reagents and plastics that could be potentially harmful in humans. A safety assessment of BM-MNC cells prepared using density gradient centrifugation should be done in order to ensure that the finished product do not contain any substance or impurity that can have an adverse effect in the patient.

BM-MNC should be free from adventitious microbial that could originate from the starting or raw materials or adventitiously introduced during the manufacturing process. In any case, a thorough testing must be performed at the level of finished product in compliance with the methodologies described in the EU or United States Pharmacopoeia (USP), in particular for endotoxin content, sterility and cell potency.

Endotoxins are lipo-polysaccharides from gram-negative bacteria and are the most common cause of toxic reactions resulting from contamination with pyrogens: the absence of bacterial endotoxins in a product implies the absence of pyrogenic components, provided the presence of non-endotoxin substrates can be ruled out. Endotoxins can be detected by using the Limulus amoebocyte lysate (LAL) test; unfortunately, it may be masked by factors interfering with the reaction between the endotoxins and the LAL. As a consequence, the suitability of the regents and materials used and the product itself has to be established. The endotoxin limit that can be accepted in a product is based on the route of administration (intravenous or intrathecal), the threshold pyrogenic dose and volume of the injected product. Some endotoxin limits have been calculated and can be found in the Pharmacopoeia; for non-compendial items and new drugs, the endotoxin limit should be calculated by the user. The Maximum Valid Dilution (MVD) provides an upper bound for dilution that still provides for endotoxin detection at the endotoxin limit. To determine if any interfering characteristics exist, each LAL assay must have a positive product control (PPC) to ensure that endotoxin would be detected if it were present in the sample.

Potency is the quantitative measure of biological activity based on the attribute of the product, which is linked to the relevant biological properties. The assay demonstrating the biological activity should be based on the intended biological effect which should ideally be related to the clinical response. Basically, two types of potency assays can be envisioned: *in vitro *assays using cell systems and *in vivo *assays using animal models. As concerning the use of bone marrow mononucleated cells in cardiac repair, the importance of characterizing the functionality of injected cells was recently pointed out [15,16]: to evaluate the functional activity of the cells obtained after density gradient centrifugation, authors purposed both *in vitro *and *in vivo *assays. Cells were evaluated for hematopoietic colony-forming unit (CFU), and assessment of mesenchymal stem cells colonies. Furthermore, based on the observation that the migratory capacity of bone marrow mononucleated cells predicts the functional improvement after cell transplantation in a hind limb ischemia model [17] and in humans [18], authors purposed the assessment of the migration capacity of the cells. At the moment there is no consensus in establishing acceptance criteria for the migration capacity of BM-MNC in cardiac regeneration.

Cell migration and cell invasion assays measure the ability of certain cell types to move through a porous membrane toward a chemoattractant or growth factor. In contrast to cell migration through an open pore, cell invasion through an occluded pore is dependent on active enzymatic degradation of the matrix barrier. The Matrigel Matrix consists of laminin, collagen IV, entactin, and various growth factors to mimic the basement membrane. Endothelial cells express proteases MMP 2 and 9, which actively digest the matrix. At the end-point of the assay, invasive cells appear on the underside of the porous membrane and can be quantified.

Guidelines for sterility testing of biologics is addressed in the various worldwide pharmacopeias and in Section 21 of the Code of Federal Regulations (CFR), International Conference on Harmonisation (ICH) and Food and Drug Administration Points to Consider documents. ATMP manufactured under GMP conditions require sterility testing performed under GMP guidelines. There are two common types of sterility test methods: the membrane filtration method that requires the test article to first pass through a size exclusion membrane capable of retaining microorganisms and the direct inoculation method requires the sample to be inoculated directly into test media. For the latter, sample is incubated for 14 days in the test media. It is important to determine if the ATMP under testing contains elements able to interfere with the growth of microorganisms within the growth media used for the assay.

Aim of this study is the validation of a commercial system (Charles River Endosafe PTS) for the determination of bacterial endotoxins in compliance with Eu Pharmacopoeia 2.6.14 (bacterial endotoxins), the validation of the sterility testing in compliance with eu Pharmacopoeia 2.6.1 (sterility) and the validation of the potency assay in an ATMP that is constituted of bone-marrow mononucleated cells used in cardiac regeneration.

## Materials and methods

Testing were performed in the quality control laboratory of the cell therapy unit of the Cardiocentro Ticino. The Laboratory is authorized and regularly inspected by the Swiss competent authorities.

### Sample Preparation

For the endotoxin testing and migration assay cells were collected after informed consent from patients enrolled in the "Swiss multicenter intracoronary stem cells study in acute myocardial infarction" (SWISS-AMI, NCT00355186). A total of 50 ml of bone marrow was aspirated into heparin-treated syringes from the posterior iliac crest under local anesthesia. Bone marrow was filtered by using a 100 μm nylon mesh (BD Falcon TM Cell Strainer, BD Biosciences), diluted 1:1 in Phosphate Buffered Saline (PBS), and BM-MNC isolated by density gradient centrifugation on Ficoll-PAQUE PREMIUM (General Electric). Cells were washed three times in PBS filtered through a 70 μm nylon mesh (BD Falcon) and then resuspended in 10 ml of 5% v/v human albumin. One ml was collected for migration and invasion assay and endotoxin testing. For the sterility testing, peripheral blood mononucleated cells were obtained from 50 ml of peripheral blood collected from patients immediately after an acute myocardial infarction (AMI) subjected to standard pharmacological therapy.

### Cell Characterization

For the immunophenotype, bone marrow and BM-MNC cells were stained in quadruplicate with anti CD45 FITC (Beckman Coulter, USA), anti CD34 PC7 (Becton Dickinson, San Jose, USA), anti CD133 PE (Miltenyi, Bergisch-Gladbach, DE) and with 7-AAD (Beckman Coulter, USA) for the cell viability test. Death cells were excluded from the analysis. Analyses were performed using a Cytomics FC 500 flow cytometer (Beckman Coulter) acquiring at least 100.000 events. Isotype-matched murine FITC, PC-7, and PE conjugated immunoglobulins were used as controls. Cell phenotype was determined by using an ABX Micros 60 (Horiba Diagnostics, France).

### Migration and Invasion Assay

**Figure 1:**
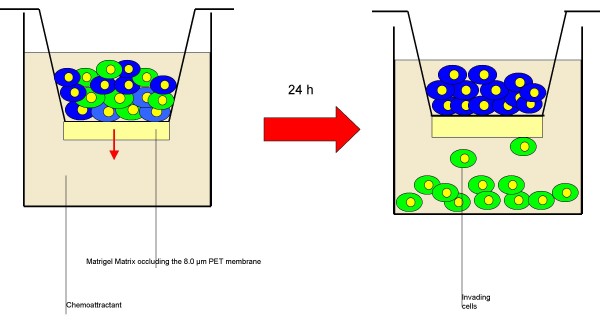
**Schematic representation of the invasion assay**. BM-MNC cells were resuspended in 5% v/v human albumin and placed in the upper part of a modified Boyden chamber Matrigel invasion chamber. The chamber consist of a 24-well cell culture insert with an 8 μm pore size PET membrane, uniformly coated with Matrigel matrix. The matrix provides a barrier to non-invasive cells while presenting an appropriate protein structure for invading cells to penetrate before passing through the membrane. The chamber was then placed in a 24-well culture dish containing 500 μl of Endocult basal medium supplemented with Endocult single quots (Stemcells technologies, Vancouver, Canada) and 20% fetal calf serum. After 24 h of incubation transmigrated cells were counted.

A total of 1 × 10^6 ^BM-MNC collected from acute myocardial infarction patients subjected to standard pharmacological therapy were resuspended in 500 μl of 5% v/v human albumin. For the migration assay, cells were placed in the upper part of an 8.0 μm untreated polyethylene terephthalate membrane 24-well cell culture insert (Becton Dickinson, CA). For the invasion assay cells were placed in the upper part of a modified Boyden chamber Matrigel Invasion Camber (BioCoat Matrigel invasion chamber, Becton Dickinson, CA): the chamber consist of a 24-well Cell Culture insert with an 8 μm pore size PET membrane, uniformly coated with Matrigel Matrix. The matrix provides a barrier to non-invasive cells while presenting an appropriate protein structure for invading cells to penetrate before passing through the membrane. Both chambers were then placed in a 24-well culture dish containing 500 μl of Endocult Basal Medium supplemented with Endocult Single Quots (Stemcells Technologies, Vancouver, Canada) and 20% fetal calf serum (Figure 1). After 24 hours of incubation at 37°C, 5% v/v CO_2 _transmigrated cells were counted. Assays were run in duplicates.

### Endotoxin Testing

#### Description of the PTS Endosafe system

The Endosafe portable test system is based on the kinetic chromogenic LAL-test that is based on the cleavage of a synthetic substrate by an enzyme produced in the reaction of the lysate in the presence of endotoxin. The system consists of LAL reagents and endotoxin controls in the form of a single-use polystyrene cartridges. The cartridges are potency tested, spike recovery is performed and the calibration code is determined. The calibration code contains the cartridge test parameters that were determined during potency testing as well as the archived curve for that batch of cartridges. The color intensity developed is proportional to the endotoxin concentration. Each cartridge consists of two sample channels and two spiked channels, consistent with current Pharmacopoeia guidance for licensed quantitative LAL methods. Each reservoir contains a specific amount of LAL reagent, synthetic chromogenic substrate, control standard endotoxin (CSE) and buffers uniformly embedded in the cartridge. The cartridge is inserted into a dedicated reader and 25 μL of the prepared sample are dispensed into the four reservoirs. The reader draws, mixes and incubates the sample with the various reagents at programmed time intervals before transferring it to the optical chambers. The portable spectrophotometer then monitors the change in the optical density and calculates the endotoxin level based on the resulting kinetic values. Cartridges with 5-0.050 EU/mL sensitivity were used in this study. Results are automatically multiplied by the dilution factor entered into the Endosafe system. With the correct dilution the unit achieves results in approximately 15 min.

#### Preparation of the inhibition/enhancement test and preparation of the cell therapy product dilution series

The calculated MVD and endotoxin limit for the ATMP were 780× and 39 EU/ml respectively. The inhibition/enhancement test was done by using the Charles River R+D Inhibition/Enhancement cartridges (range 5-0.05 EU/ml ) and by testing the cell product undiluted and diluted in pyrogen-free water as follows: 1:10; 1:100; 1:500; 1:700; 1:780.

This preliminary assay was performed with the aim to find the dilution where the spiked endotoxin can be detected without inhibiting or enhancing the test. Once prepared, the cartridge was inserted in the Endosafe PTS and loaded with 25 μl of the solution in each well. Results were scored after 20 minutes of incubation at 37°C.

The ATMP was diluted in LAL reagent water (Charles River ) to 1:10 and 1:100 in pyrogen-free tubes and then loaded in the system. All the tubes, water and pipette-tips were pyrogen-free certified.

### Sterility Testing

Sterility testing was carried out under aseptic conditions regularly monitored by appropriate sampling of the working area and by carrying out appropriated controls as specified in on GMP documents.

#### Growth promotion test (GPT)

Sterility of the culture media Fluid thyoglicollate medium (FTM) and soya-bean casein digest medium (TSB) used for the culture of anaerobic and fungi/aerobic bacteria (THIOC-T and TSB-T, bioMerieux SA, Switzerland) was performed by incubating two vials of medium for 14 days at 32.5°C and 22.5°C respectively. Growth promotion test was performed by inoculating FTM media with 10-100 colony-forming units (UFC) of *Bacillus subtilis *ATCC 6633; *Staphylococcus aureus *ATCC 6538; *Pseudomonas aeruginosa *ATCC 9027; *Clostridium sporogenes *ATCC 19404 and TSB media with 10-100 UFC of *Candida albicans *ATCC 10231; *Aspergillus niger *ATCC 16404 and *Bacillus subtilis *ATCC 6633 (all from Quanti-Cult, Remel, Lenexa, KS). Media were incubated as described for five and three days respectively. Culture plates were inoculated in parallel in order to check the viability of the micro-organisms. Testing was also performed by using the following bacterial strains isolated from bioburden in clean room: *Staphilococcus epdermidis 1*, *Micrococcus lylae *and *Sphingobacterium multivorum*. All testing were performed in duplicate. Bacterial identifications were performed by Gram-staining and by using the mini API detection system (bioMerieux SA, Switzerland). The ID32 and ATB test strips were used for the strain identification (bioMerieux SA, Switzerland).

#### Validation test

Validation was performed by direct inoculation of TSB and FTM media with 1% of the total volume of the product under validation as stated in European Pharmacopoeia (2.6.27). For the latter, 500 μl of whole blood and 100 μl of the BM-MNC were inoculated together with 1-10 UFC and 10-100 Colony-forming units of the bacterial strains used in the growth promotion test and incubated as above described. A growth promotion test was performed as a positive control. If clearly visible growth of micro-organisms is obtained after incubation in presence of blood and the ATMP, the product possesses no antimicrobial activity under the conditions of the test, and the sterility may be then carried out without further modification.

### Data Analysis

For the endotoxin testing, a test result was considered valid when the percentage of spike recovery was between 50% and 200% with a coefficient of variation less than 25%.

For the sterility testing, the detection limit represent the lowest bacterial concentration in the inoculums that the system can evidence. The specificity of the system represent its ability to detect the single micro-organism in the inoculums and the detection limit represent the lowest micro-organism number in the sample that the system can detect. The robustness of the system represent its ability to obtain identical results when using different products, medium from different lots in different working days.

For the invasion assay, data were expresses as the percent invasion through the Matrigel matrix and membrane relative to the migration through the 8.0 μm untreated Membrane (invasion index). The Assay was considered positive when at least ≥10% of the inoculate cells maintain their invasion capacity.

## Results

### Cell phenotype

**Figure 2:**
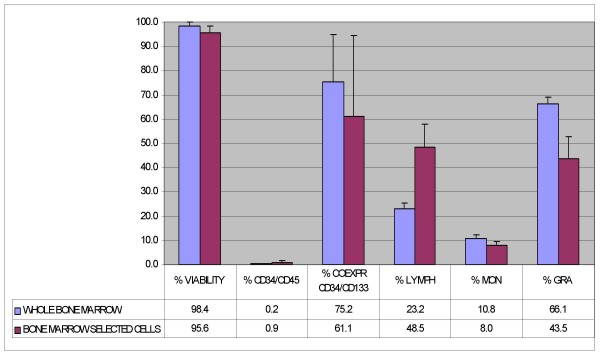
Phenotypical analysis of whole bone marrow cells and after density gradient centrifugation (bone marrow selected cells) (n = 4).

Cell phenotype of whole bone marrow and after density gradient separation are reported in Figure 2 (mean ± SD, n = 4).

### Endotoxin testing

Testing was performed on three BM-MNC obtained from different patients in three different days. Patient were subjected to standard pharmacological treatment for acute myocardial infarction. The mononucleated cells concentration in the samples were 18.0 × 10^6^/ml; 15.2 × 10^6^/ml and 16.2 × 10^6^/ml respectively (16.5 ± 1.2 × 10^6 ^mean ± SD) with a pH of 6.5.

Results of the inhibition/enhancement test are reported in Table 1. Based on the obtained results, the 1:10 and 1:100 dilutions were selected for the validation assay. An invalid value, based on acceptance criteria, was observed in the first run for the 1:10 dilution. The results of the validation assay are reported in Table 2.

**Table 1:** Results of the inhibition/enhancement test

**SAMPLE DILUTION**	**SPIKE RECOVERY**
Undiluted	162%
1:10	53%
1:100	113%
1:500	132%
1:700	120%
1:780	98%

**Table 2:** Results of the validation assay

	**1:10 DILUTION**						**1:100 DILUTION**					
	**1^st ^run**		**2^nd ^run**		**3^rd ^run**		**1^st ^run**		**2^nd ^run**		**3^rd ^run**	
Spike recovery (PPC)	122	119	121	115	76	95	143	178	176	163	142	183
PPC CV (%)	14.1	18.7	15.8	4.0	7.3	8.0	15.0	2.7	0.7	7.2	7.4	9.6
Sample CV	3.5	1.5	0	0	0	0	0	0	0	0	0	0

Sample result (EU/mL)	<0.532	<.513	<0.500	<0.500	<0.500	<0.500	<0.500	<0.500	<0.500	<0.500	<0.500	<0.500

### Sterility testing

Testing was performed on three whole peripheral blood and the derived mononucleated fractions from different patients in three different days. Patient were subjected to standard pharmacological treatment for acute myocardial infarction. The white blood cell concentration in the mononucleated fraction were 13.0 × 10^6^/ml; 12.2 × 10^6^/ml and 15.2 × 10^6^/ml respectively (13.5 ± 1.6 × 10^6 ^mean ± SD) with a pH of 6.5.

For the growth promotion test at the end of the incubation period, clearly visible growth of micro-organisms was observed and identity confirmed for all bacterial strains.

As concerning the strains isolated from bioburden, *S. epidermidis 1 *growth in both TSB and FTM media at both concentrations whereas *M. lylae *and *S. multivorum *growth at both concentrations in TSB medium only. For the validation test, all the FTM cultures resulted to be positive after 3 days at both the concentration tested. For TSB cultures, *Mycetes *were positive after 5 days and *B. subtilis *after three. The detection limit of the system was then established in 1-10 colonies. At the end of the incubation period, subcultures in agar plates were performed for all the microbial growth: all the identifications confirmed the starting inoculum confirming the robustness of the system.

### Migration and invasion assay

**Figure 3:**
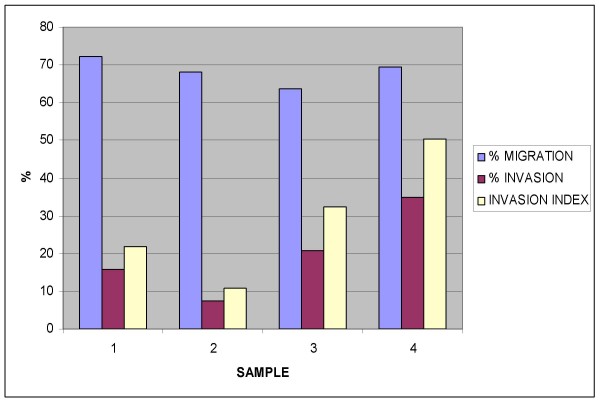
**Migration and invasion assay results for bone marrow derived mononucleated cells**.

A total of four assays were performed in different days. For all the samples a significant invasion index was observed: 28.89 ± 16.82% (mean ± SD). Complete results are reported in Figure 3.

## Discussion

Cellular therapy is an emerging field in medicine; all the stem cell medicinal products must be in compliance with principles and guidelines of good manufacturing practice in respect of medicinal products and investigational medicinal products for human use. When any new preparation or method of preparation is adopted, steps should be taken to demonstrate its suitability for routine processing: the defined process, using the materials and equipment specified, should be validated in order to produce cells of the required quality.

For certain ATMP that must be administered immediately and that cannot be cryopreserved without damaging the cell viability and quality, the availability of rapid testing method for endotoxin and sterility testing is fundamental.

For the latter, traditional methods, including kinetic chromogenic, kinetic turbidimetric and gel-clot LAL assay systems, have been widely used in the pharmaceutical industry. Unfortunately, all of these methods are time-consuming (several hours) and become problematic if time-sensitive ATMPs products must be immediately released. In the present paper, we have demonstrated that the PTS endosafe system can be validated for the endotoxin testing of BM-MNC in compliance with European and United States Pharmacopoeia. The time required by the system was approximately 15 min, making it particularly useful as an immediate release testing, where the aim is to prepare and administer the product within a short time period.

Sterility testing is regulated by USP 21CFR610.12 and by Eu Pharmacopoeia 2.6.1. We have successfully validated the sterility testing of a mononucleated cell preparation: the sensitivity of the system for the ATCC and bioburden bacterial strains here considered was 1-10 UFC in the inoculums and cultures were positive after approximately 48 hours of incubation.

Recently, a rapid microbiological control strategy for cellular products has been issued in EU and USP Pharmacopoeias based on the use of rapid detection systems as the BacT/Alert 3D (bioMerieux, Durham, USA) or the Bactec (Becton Dickinson, Franklin Lake, USA). Those systems are in general non destructive, allowing a faster detection when compared to TSB/FTM testing, and products can be released after 7 days. Unfortunately, the microbial growth of certain bacterial strains in those systems is still controversial; as a consequence, those method should be strictly validated both using the prescribed ATCC strains and by using bioburden isolates.

All biological products must meet prescribed requirements of safety, purity and potency and no lot of any licensed product may be released by the manufacturer prior to the completion of tests for conformity with standards applicable to such product, including potency. The current regulations allow for considerable flexibility in determining the appropriate measurements of potency that is necessary for product characterization testing; however, the complexity of an ATMP product can present significant challenges in establishing a potency assays.

The migration assay of BM-MNC in response to endothelial growth factors, seems to correlate with the beneficial effects of the cell infusion after myocardial infarction [15,16]: this assay has been then purposed as a quantitative biological measure for the activity of the product related to its specific ability to achieve the given result. In particular, has been suggested that the correlation between the "in vitro" data and the clinical efficacy may be obtained by analyzing the outcomes from controlled clinical studies [19,20]. In addition to the migration assay, here we describe the use of the invasion assay as a potency testing for BM-MNC cells: we purpose to define as a minimal criteria to establish cell potency in cardiac regeneration, the obtainment of an invasion index not less than 10%. We are aware that the cell migration and invasion results "in vitro" should be correlated with the "in vivo" effect of the cells and this must be addressed both in a suitable animal model and during a controlled clinical trial of acute myocardial infarction.

Basic and clinical scientists, as well as scientists working in the biotechnology and pharmaceutical industries, need an increased awareness of the questions that must be answered before a stem-cell-based product can be used clinically. Unlike pharmaceutical products, many stem-cell-based products may originate in academic laboratories where researchers are unfamiliar with the applicable regulations. As new stem-cell-based therapies are developed, the regulatory framework is likely to evolve. Meanwhile, existing regulations pertaining to biologic products and human cells, tissues, and cellular and tissue-based products provide an appropriate structure for ensuring the safety and efficacy of the next generation of stem cell-based medicinal products. As they conduct research on stem cells, scientists should be aware of the relevant regulations and their likely application to this products.

## Competing interests

The authors declare that they have no competing interests.

## Authors' contributions

GA wrote the manuscript, SS and VLC performed the experiments, DS performed the sample collections as co-investigator of the Swiss Ami clinical Trial, MG performed literature search, GS and TM participated in study design and coordination. All the authors read and approved the final manuscript.
